# Supplementary material for: Epidemiological dynamics of an urban Dengue 4 outbreak in São Paulo, Brazil
Source: PeerJ. 2016 Apr 5;4:e1892. doi: 10.7717/peerj.1892 (PMC4824887; doi:10.7717/peerj.1892)
Supplement: Supplemental Information 1 — Notes: *The Birth–death skyline (BDSKY) is implemented in Beast v2.3.1 (Bouckaert et al., 2014). The BDSKY parameterization consisted of three parameters that can be estimated in a piece-wise manner over different intervals: (i) the effective reproduction number (R), (ii) the become un-infectious rate (y) and, (iii) the sampling proportions (s). For parameters R and y (which is equivalent to the inverse of the infectious period) we used lognormal distributions following the same rationale behind the prior of the incidence time series analysis (see main text). For parameter s, we used a Beta prior that also considered smaller proportions than the observed/sampled infections (0.2%). This is because dengue infection is characterized by an iceberg effect, in which most cases are asymptomatic with documented symptomatic-to-unapparent ratios as large as 1:18 (Balmaseda et al., 2010; Endy et al., 2011; Yap et al., 2013). We constrained s and y to be constant through time but R was estimated in a piece-wise manner over six different intervals. **Truncated normal distributions used to account for uncertainty on the serial interval distribution. [file peerj-04-1892-s001.docx]

|  | Prior | Distribution | Parameter | |
| --- | --- | --- | --- | --- |
| Substitution model (Tamura-Nei, TrN) | Rates | Lognormal | Mean= 1.0 | Standard deviation = 1.25 |
| Sites model | Proportion of Invariants | Uniform | Bounds (0,1) | |
| Clock models | Uncorrelated lognormal relaxed clock mean | Uniform | Bounds (0,∞) | |
|  | Uncorrelated lognormal relaxed clock standard deviation | Gamma | Alpha=0.5 | Beta=0.4 |
| Birth-death epidemiological model* | Reproduction number | Lognormal | Mean = 1.0 | Standard deviation = 1.0 |
|  | Become un-infectious rate | Lognormal | Mean= -2.0 | Standard deviation = 0.5 |
|  | Sampling proportion | Beta | Alpha= 2.0 | Beta= 20.0 |
|  | Origin | Uniform | Bounds (0,∞) | |
| R using incidence time series | Reproduction number | Gamma | Mean = 4 | Standard deviation = 2.8 |
|  | Serial Interval Distribution | Shifted gamma (+1) | Mean = 2.5 | Standard deviation = 1.75 |
|  | Average mean serial interval (Mean=2.5)** | Normal | Bounds (1.25-3.75) | Standard deviation = 0.5 |
|  | Average standard deviation of the serial interval (Mean=1.75)** | Normal | Bounds (0.25-3) | Standard deviation = 0.75 |
