# Supplementary figures and images for: Epidemiological dynamics of an urban Dengue 4 outbreak in São Paulo, Brazil
Source: PeerJ. 2016 Apr 5;4:e1892. doi: 10.7717/peerj.1892 (PMC4824887; doi:10.7717/peerj.1892)

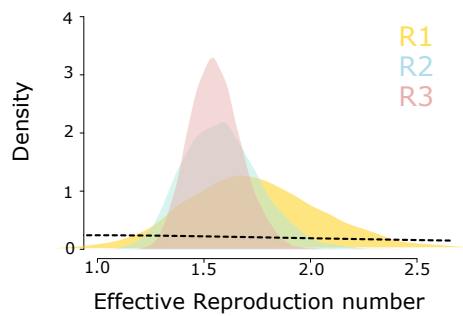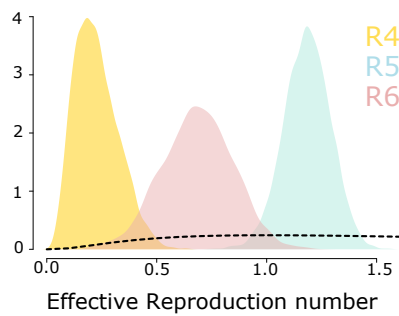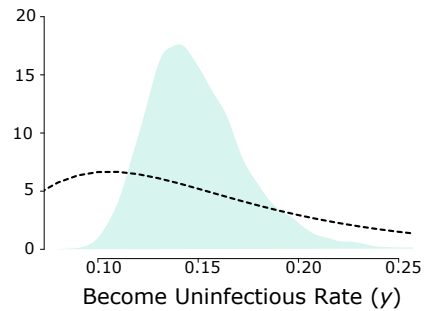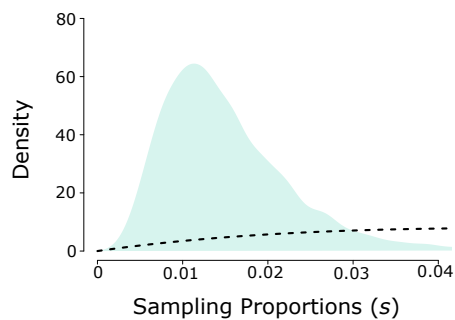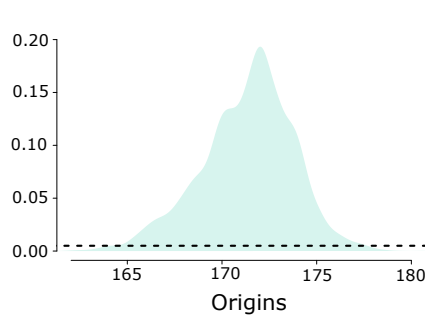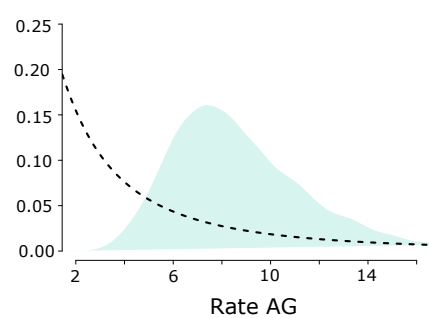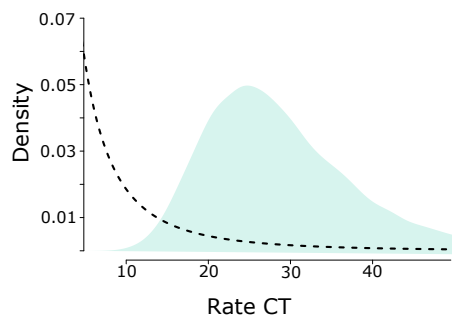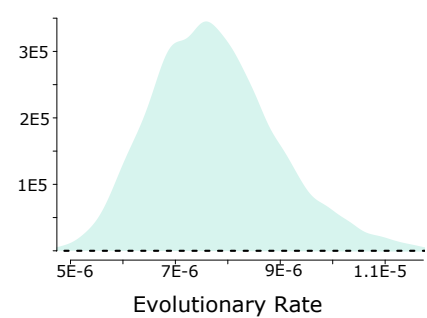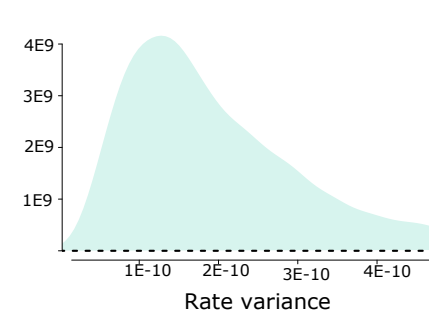

- - - - - Prior Distribution

Supplement: Supplemental Information 2 — The analyses were done with the 286-georeferenced samples. Colored regions correspond to prior densities; areas below the dashed lines correspond to prior densities. [file peerj-04-1892-s002.pdf]
